# Relation between metacognitive strategies, motivation to think, and critical thinking skills

**DOI:** 10.3389/fpsyg.2023.1272958

**Published:** 2023-12-04

**Authors:** Carlos J. Ossa, Silvia F. Rivas, Carlos Saiz

**Affiliations:** ^1^Educations Science Department, University of the Bío Bío, Concepción, Chile; ^2^Psychology Faculty, University of Salamanca, Salamanca, Spain

**Keywords:** critical thinking, structural models, cognition, motivation, pedagogy

## Abstract

Critical thinking is a complex reasoning skill, and even though it is hard to reach a consensus on its definition, there is agreement on it being an eminently cognitive skill. It is strongly related with reflective and metacognitive skills, as well as attitudinal or motivational aspects, although no model has yet been able to integrate these three elements. We present herein the preliminary results of a study seeking to establish these relations, in a sample of Chilean university students. 435 students from three universities participated, of which 88 were men, 333 were women, and 14 did not indicate their gender. Their ages ranges between 18 and 51 years old (*M* = 21, SD = 3.09). Three instruments were applied, one to measure metacognitive strategies, one to measure motivation to critical thinking, and a third to measure critical thinking skills. The relation was analyzed via structural equations. The results show a positive, strong, and significant relation between metacognition and motivation to think. However, only a weak significant relation was observed between motivation to think and critical thinking, and no direct relation was found between metacognition and critical thinking. We hypothesize a significant but moderate relation between the variables, where metacognition influences motivation to think, which in turn influences critical thinking skills. Factors are discussed which could negatively affect the studied relations, as well as the importance of generating integrated models between the three variables, as they would show a theoretical and empirical link.

## Introduction

Critical thinking is a relevant topic for the 21st century, highlighted by Unesco as one of the skills to develop among students to properly face the challenges of this century (Scott, 2015). Despite its importance for human development, its implementation in educational curricula has been difficult to carry out, both at the level of school systems and in higher education systems (Ossa et al., 2018; Silva Pacheco, 2019).

This difficulty of incorporating critical thinking into the educational process may be related with the complexity of the task. On one side, there is discussion as to whether the process can be taught as a skill, or whether it is more of a facet of thinking which can only be stimulated in a concrete way (Saiz, 2017). Building on this factor, the complexity of the matter is also expressed in the attempts at defining the process, since there are various definitions of critical thinking. These definitions present different natures, ranging from only cognitive reasoning processes; cognitive and metacognitive processes; cognitive, metacognitive and attitudinal processes; and finally, cognitive, metacognitive, attitudinal, and social agency processes (Montero, 2010; Rivas and Saiz, 2012; Ossa and Díaz, 2017; Saiz, 2017).

As society and socio-cultural challenges have become more complex, it is necessary to adopt more complex perspectives on human processes. Critical thinking perspectives which help integrate diverse processes could be more pertinent for the effective development of this skill among people (Paul and Elder, 2003).

Critical thinking has been linked to different skills, both cognitive and non-cognitive, for example, problem solving, scientific reasoning, motivation, metacognition, and now ultimately creativity (Saiz and Rivas, 2008; Tamayo-Alzate et al., 2019; Halpern and Dunn, 2021; Muñoz and Ruiz, 2022; Santana et al., 2022). Of these skills, problem solving has been incorporated as a constituent element of critical thinking in some models; Likewise, motivation and metacognition are closely related factors and it has been proposed that they are satellite skills for critical thinking processes (Valenzuela and Nieto, 2008; Rivas and Saiz, 2011; García, 2022), although no empirical information has been shown to clearly demonstrate this. The objective of this paper is precisely to show the relationship between motivation to think and metacognition with critical thinking, in order to contribute to what is proposed.

## Critical thinking, motivation, and metacognition

Even when critical thinking is a broadly used concept in the academic and educational world, with a wide range of studies in the last decade, it continues to be a difficult phenomenon to conceptualize and to create little consensus (Ossa et al., 2016; Saiz, 2017; Díaz et al., 2019).

It is conceptualized as a cognitive mechanism which filters information about the ideological intentions accompanying said information, via continual questioning of knowledge production practices, and the recognition of its different perspectives (Yang and Chung, 2009; Montero, 2010).

It is a type of thinking oriented toward data and action, in a context of solving problems and interacting with other people (Daniel and Auriac, 2012; López, 2012). Critical thinking is self-directed, self-disciplined, self-regulated and self-corrected. It involves undergoing rigorous standards of excellence and a conscious dominion of its use. It also implies effective communication and the development of problem solving skills (Saiz and Rivas, 2008, 2012, 2016).

Critical thinking is characterized by generating higher-level cognitive processing in people, centered on the skills of reflecting, comprehension, evaluation and creation. It therefore requires high intellectual development. However, it is also a skill which can be developed, since there are no important differences between people with average and high intellectual levels with regards to developing critical thinking (Sierra et al., 2010).

Since critical thinking is a high-level cognitive process, and the ability to generate an elaborated thought, a close relation has been proposed with elements which are not considered merely cognitive, including metacognition (Rivas et al., 2022). Metacognition is a reflective process which helps deepen thought, regulate, and generate consciousness about thought (Tamayo-Alzate et al., 2019; Drigas and Mitsea, 2020). It has been worked on as both a reflective process of self-knowledge, and as a skill which helps develop other cognitive processes including memory, learning, or even intelligence, since different levels of application can be established in its use (Drigas and Mitsea, 2021).

There is evidence that metacognitive strategies can influence critical thinking and its components. For one, it improves the use of metacognitive strategies due to intervention in critical thinking. It also improves the use of critical thinking with metacognitive strategies in interventions done with psychology students at universities (Ossa et al., 2016; Rivas et al., 2022). Significant and positive relations have also been found between critical thinking and metacognitive consciousness among medical students, although not for regulation and knowledge tasks (de la Portilla Maya et al., 2022).

In this way, we can observe a relative influence on the way that people think about thinking, since metacognition supports decision making and final evaluation about strategies to resolve problems (Rivas et al., 2022).

Some authors also indicate the presence of another non-cognitive component in critical thinking, which is disposition or motivation (Facione et al., 2000; Saiz and Rivas, 2008; Marin and Halpern, 2011; Valenzuela et al., 2014; Halpern and Dunn, 2023). This component is fundamental to achieve this skill, since even when the indicated cognitive functions are available, if people either lack the desire to apply critical thinking or deem it inconvenient to do so, critical thinking will not be adequately manifested (Valenzuela and Nieto, 2008; Valenzuela et al., 2014).

This non-cognitive element is based on human attitudes or motivations which complement the use of critical thinking, allowing it to be better developed, since they drive personal improvement (Boonsathirakul and Kerdsomboon, 2021). The factors presented as facets of a disposition toward critical thinking include seeking truth, open-mindedness, being analytical, systematicity, curiosity, self-confidence and maturity (Facione, in Boonsathirakul and Kerdsomboon, 2021).

However, considering these non-cognitive elements as dispositions of a being also involves assuming certain personality traits or dimensions of values which cannot always be adequately measured. They should thus be considered more as motivational aspects, since they could be better defined and with a greater possibility of modification, given that they are more related with behavioral and perceptual elements (Valenzuela et al., 2014, 2023). From this perspective, we understand that non-cognitive components are based on the expectations and value given to the task. In this way, we establish a direct and causal relation between motivation and critical thinking, where the former explains critical thinking development by between 8 and 17%, according to the instrument used to measure it (Valenzuela et al., 2023).

In this way, promoting motivational aspects is a relevant factor for developing cognitive and metacognitive processes, since complex processes are exhausting and require a high and constant investment of cognitive and emotional factors (Valenzuela and Nieto, 2008; Valenzuela and Saiz, 2010; Gaviria, 2019; Nieto-Márquez et al., 2021).

Finally, a relative relation has been noted between motivational processes and metacognitive strategies. Correa et al. (2019) performed an evaluation among Chilean high school students about the use of metacognitive strategies and motivation to critical thinking in bias recognition. They found a positive, significant, and medium-intensity correlation (*r* = 0.50, *p* < 0.001) between both variables, which indicates that cognitive and non-cognitive factors have a relevant link for human thought.

With the aforementioned background, we can hypothesize the existence of a significant and positive relation between critical thinking, metacognitive strategies, and motivation to think critically; that motivation to think directly affects critical thinking; and those metacognitive strategies are related with both variables.

In this article it will be showed preliminary results from this relation, presenting a relational model based on structural equations which would allow for establishing direct and mediated relations between said variables.

## Method

A correlational study was done via structural equations.

### Participants

435 students from pedagogy majors at three Chilean universities participated in the study. Of these, 88 were male (20.2%), 333 were female (76.6%), 7 were students of unidentified gender (1.6%), and 7 did not respond (1.6%). Students’ ages fell between 18 and 51 years (*M* = 21, SD = 3.09). The careers to which the students belong are in the area of pedagogy, in specialties of mathematics (22%), history (8%), science (15%), special education (15%), and early childhood education (40%).

### Instruments

For this study, a battery with three instruments was applied:

1. Metacognitive strategy questionnaire from O’Neil and Abedi, adapted into Spanish by Martínez (2007). This measure metacognitive strategies applied to different academic tasks. There are 20 items organized into three dimensions: self-knowledge (referring to metacognitive consciousness), self-regulation (referring to metacognitive control), and evaluation (referring to global task evaluation). Results are recorded with a Likert-type scale of 5 choices (0 to 4 points). This instrument has been applied to Chilean university students and shown adequate reliability indicators. The global Cronbach’s *α* was 0.87, and for the dimensions it was between 0.62 and 0.65 (Correa et al., 2019).
2. Critical thinking motivation questionnaire from Valenzuela, measuring the intention of applying thinking to knowledge tasks, based on personal expectations and the value of the task. It contains 19 items organized into 5 dimensions: Expectation (*α* = 0.774), Importance (*α* = 0.770), Cost (*α* = 0.775), Utility (*α* = 0.790) and Interest (*α* = 0.724). Its results are recorded based on a Likert-type scale with 5 alternatives (0–4 points). It has been applied to Chilean university students with strong reliability indicators. The global Cronbach’s *α* was 0.92, and the values for its dimensions ranged from 0.69 to 0.83 (Valenzuela and Nieto, 2008; Correa et al., 2019).
3. Critical thinking task test from Miranda, adapted by Palma Luengo et al. (2021). This measured the capacity to apply cognitive critical thinking processes to socio-scientific topics. It contains 15 items organized into three dimensions: inquiry (referring to identifying useful information), analysis (referring to the decision to use pertinent and reliable data), and arguing (referring to providing arguments with useful and reliable data). Its results are recorded with a sequence of scores ranging from 0 to 3 points, based on a performance rubric. It has been applied to a sample of Chilean university students with moderately adequate reliability indicators. The overall Cronbach’s α was 0.67, with moderately low values in its dimensions ranging from 0.47 to 0.60 (Palma Luengo et al., 2021).

Three metacognition questions were incorporated into this instrument to reflect on the tasks being done, one for each dimension (e.g., *How are you so confident about knowing how to do the activity?*). Two questions about motivation to thinking were also included, in the middle and at the end of the test, seeking to analyze whether there was a disposition to answer a question in a more voluntary form (e.g., *Do you want to finish the test here or do you want to continue to delve deeper into the topic?*). The overall Cronbach’s α was 0.78 (five dimensions), and the values were moderately adequate within these dimensions (0.54 for metacognition and 0.73 for motivation).

### Procedures

We made contact with the directors of the pedagogy majors at three different universities, coordinating the process and determining the courses to consider. After this, a talk was carried out in each course, inviting students to participate in the study. Written informed consent was incorporated into the survey, indicating the study objectives and describing the anonymous and voluntary nature of participation. Open consultations were made about participation in applying the surveys, applying the battery of instruments only to those who wished to participate.

After answering the instruments, the data was emptied into a digital database and analyzed with SPSS v.27 and RStudio software. For data analysis, we used inferential and multivariate statistics. For all inference effects, a 5% significance threshold has been considered. In the structural models, we applied formats from Partial Least Squares (SEM-PLS).

## Results

We present an application of structural equations based on partial least squares (PLS), designed to model behavioral situations and social sciences. According to Wold (1980) it is fairly flexible, since it is useful for small sample sizes and also does not require distributional assumptions for the variables, along with being useful for predictive analysis as well as theoretical confirmation. With the PLS format, there are three methodological considerations which are relevant for application: (i) choosing variable with items that effectively belong, (ii) valuing items’ reliability and validity, and (iii) properly interpreting the coefficients.

As indicated in this type of modeling, there are two sections. The first is the measurement model, where each dimension is formatively related with its items: i.e., the item contributes to the variable with a certain coefficient called weight (*w*). This factorial weight represents the weighting of the dimension regarding the latent variable which it intends to measure, so that we can expect it to have sufficient magnitude to be statistically significant.

To begin, for the Metacognition variable, the scores for Self-Knowledge (*w* = 0.67, *p* < 0.001, 95% IC: 0.41; 0.97) and Evaluation (*w* = 0.34, *p* < 0.01, 95% IC: 0.12; 0.56) are relevant for generating the latent indicator. For the Motivation variable, the scores for Expectations (*w* = 0.21, *p* < 0.05, 95% IC: 0.25; 0.62), Importance (*w* = 0.43, *p* < 0.001, 95% IC: 0.14; 0.60), and Usefulness (*w* = 0.39, *p* < 0.001, 95% IC: 0.19; 0.25) are representative when generating this indicator. For Critical Thinking, only the Metacognition indicator (*w* = 0.71, *p* < 0.05, 95% IC: 0.56; 0.86) turned out to be appropriate.

The second section of this type of models is called the structural model. It shows the causality relations between the latent variables. Schematically, we consider that a variable X is the cause of another variable Y, and an arrow will go from X to Y. For this study, the relational schematic between variables is given by the following hypothesis set:

*H1*: There is a positive effect of the Metacognition Strategy (ME) on Critical Thinking Motivation (MO).

*H2*: There is a positive effect of Metacognition Strategy (ME) on Critical Thinking (PC).

*H3*: There is a positive effect of Critical Thinking Motivation (MO) on Critical Thinking (PC).

**Figure 1:**
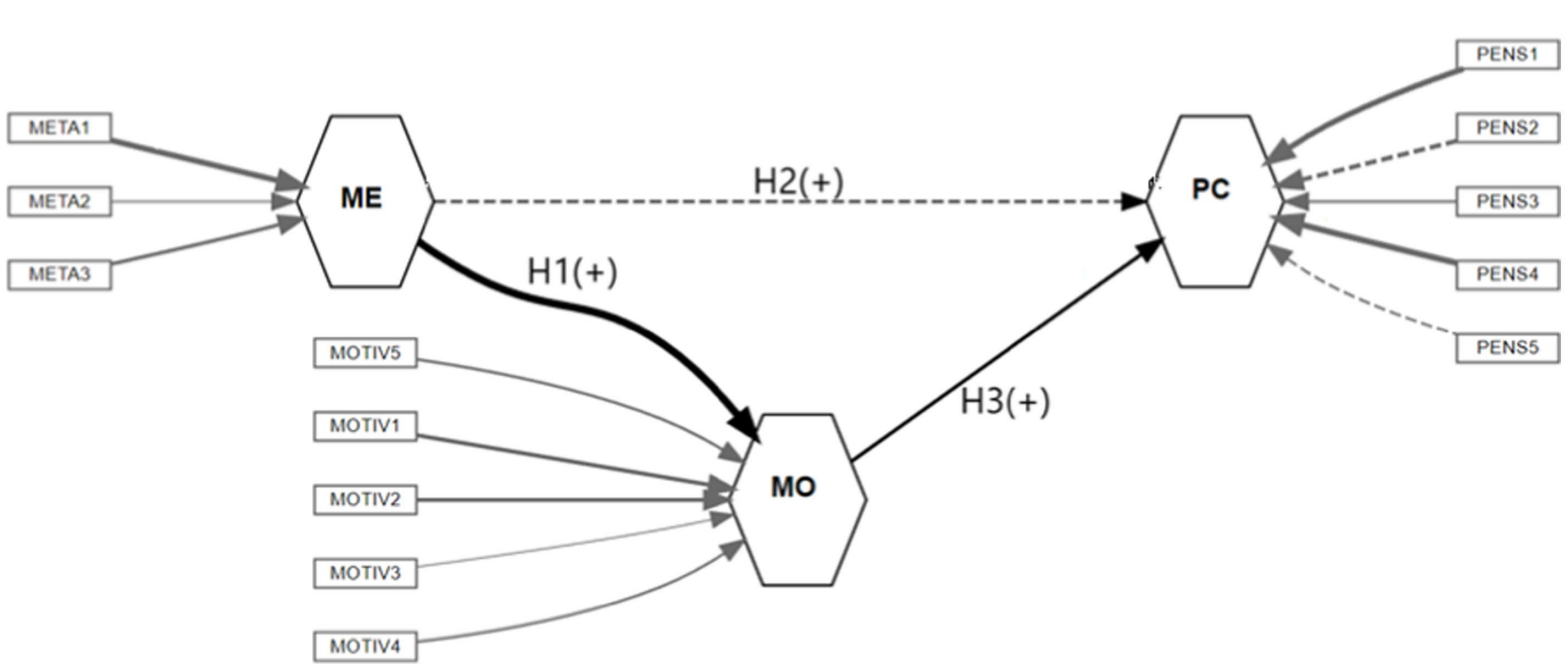
Schematic of hypothesis and effects expected. Structural equation model. Source: authors.

Figure 1 shows the hypotheses combined with their respective variables, indicating the measurement and structural models.

The empirical results from the model appear in Table 1 with their significance level.

**Table 1:** Structural equation model results.

Hypothesis	*B*	95% BIC
Metacognition		
Self-recognition → ME	0.666***	**[0.41; 0.97]**
Self-regulation → ME	0.075	[−0.19; 0.31]
Evaluation → ME	0.342**	**[0.12; 0.56]**
Motivation		
Expectations → MO	0.209*	**[0.25; 0.62]**
Importance → MO	0.425***	**[0.14; 0.60]**
Usefulness → MO	0.394***	**[0.19; 0.25]**
Cost → MO	0.019	[−0.04; 0.47]
Effort → MO	0.191	[−0.04; 0.40]
Critical thinking		
Inquiry → PC	0.653	[−0.57; 1.19]
Analysis → PC	−0.471	[−1.11; 0.47]
Arg. Comm. → PC	0.172	[−0.72; 0.84]
Metacognition → PC	0.713*	**[0.56; 0.86]**
Motivation → PC	−0.174	[−0.67; 0.60]

Hypothesis	*B*	95% BIC
*H1*: ME → MO	0.564***	**[0.49; 0.63]**
*H2*: ME → PC	−0.076	[−0.23; 0.12]
*H3*: MO → PC	0.209**	**[0.06; 0.34]**

*p < 0.05; **p < 0.01; ***p < 0.001.Bold values corresponding to statistically significant value.

**Figure 2:**
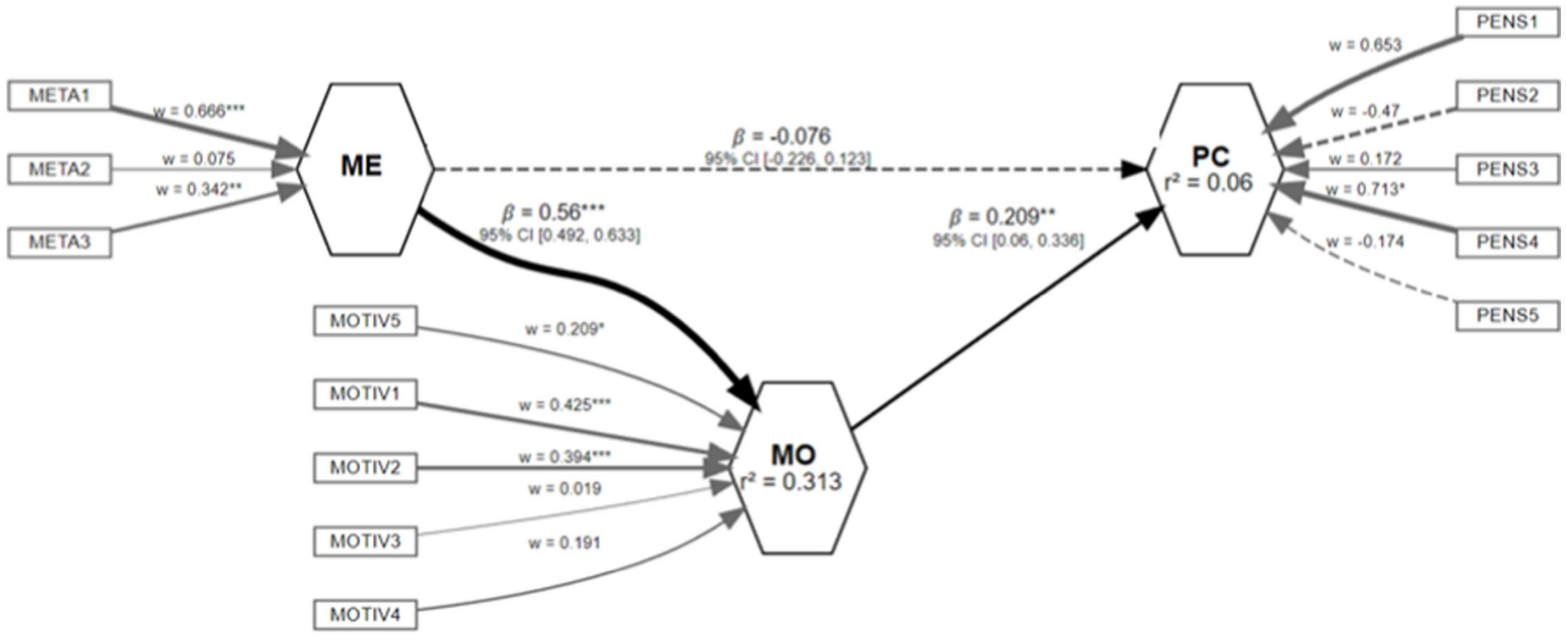
Results schematic. Structural equations model. Source: authors.

Finally, in the structural model (Figure 2), we can see the fulfillment of hypothesis H1 (*B* = 0.56, *p* < 0.001, 95% IC: 0.49; 0.63) where a greater perception of Metacognition leads to a greater level of Critical Thinking Motivation. There is also fulfillment for hypothesis H3 (*B* = 0.21, *p* < 0.01, 95% IC: 0.06; 0.34) indicating that greater levels of Critical Thinking Motivation lead to a greater level of Critical Thinking.

## Discussion

Our preliminary study results show ties between the three variables, as indicated both in theory (Facione et al., 2000; Valenzuela and Nieto, 2008; Tamayo-Alzate et al., 2019) and in other studies (Correa et al., 2019; Rivas et al., 2022; Valenzuela et al., 2023). However, we found some disparate data with regards to the latter points.

For the structural models, hypotheses H1 and H3 have been fulfilled, reporting statistically significant evidence that greater perceived Metacognition explains a greater level of Critical Thinking Motivation, a greater level of Critical Thinking Motivation implies a higher level of Critical Thinking.

One important aspect here is that a significant relation was found between motivation and critical thinking skills, which is supported by Valenzuela et al. (2023). While the value of the relation is moderate, it can be related, as presented in the aforementioned study, and may be due to the type of instrument used to measure critical thinking. One notable aspect is that the motivation question incorporated into the critical thinking task instrument had little weight within this instrument. However, this could be explained because the questions sought to consider effort for the task. Reviewing the components of the critical thinking motivation survey, the dimensions with the strongest ties were those oriented towards expectations, usefulness and importance, not effort or energy costs.

It is possible that the relationship between metacognition and motivation to think is established because, from the theoretical model used (Valenzuela and Nieto, 2008; Valenzuela et al., 2014), the expectation of the task, and its assessment of usefulness (aspects motivation), require an evaluation process (metacognitive aspect); However, this idea must be deepened and reviewed in more detail.

Considering metacognition, no direct relation was observed between the instrument used in this study to measure the metacognitive strategies of self-knowledge, self-regulation and evaluation on one hand, and critical thinking on the other. This situation goes against other studies’ findings (de la Portilla Maya et al., 2022; Rivas et al., 2022), and may be explained by the type of instrument used, which may not be sensitive to the critical thinking tasks measured by the test from Palma Luengo et al. (2021).

The relation discovered about metacognition supporting critical thinking motivation, in order to thus achieve better critical thinking, is one of the key relevant findings in this study. It implies that reflecting on oneself and tasks can generate greater expectations and evaluation for the task, which can drive better performance. These results still need more breadth and depth from further research.

This study is only a preliminary report of results, to account for the relationship between the aforementioned variables and propose that critical thinking benefits from metacognitive and motivational work. Its limitations are the fact that its objective was only empirical, in order to account for the relationship raised in studies (Valenzuela and Nieto, 2008), so the theoretical depth was less. On the other hand, there was a limited number of participating students, and only from some university majors. Likewise, it is considered that the critical thinking test that was used presents adequate reliability values overall, but with less powerful values in some of its dimensions (specifically, inquiry and motivation). It is considered necessary to replicate the study with another instrument and a larger sample to more fully support the results found.

## Data availability statement

The raw data supporting the conclusions of this article will be made available by the authors, without undue reservation.

## Ethics statement

The studies involving humans were approved by Pedro Labraña Research Unit of Bio-Bio University. The studies were conducted in accordance with the local legislation and institutional requirements. The participants provided their written informed consent to participate in this study.

## Author contributions

CO: Conceptualization, Methodology, Project administration, Writing – original draft. SR: Investigation, Supervision, Writing – review & editing. CS: Conceptualization, Methodology, Writing – review & editing.
